# Transmissible cancer and the evolution of sex

**DOI:** 10.1371/journal.pbio.3000275

**Published:** 2019-06-06

**Authors:** Frédéric Thomas, Thomas Madsen, Mathieu Giraudeau, Dorothée Misse, Rodrigo Hamede, Orsolya Vincze, François Renaud, Benjamin Roche, Beata Ujvari

**Affiliations:** 1 CREEC/UMR CNRS 5290-IRD 224-Université de Montpellier, Montpellier, France; 2 Centre for Integrative Ecology, School of Life and Environmental Sciences, Deakin University, Waurn Ponds, Victoria, Australia; 3 School of Natural Sciences, University of Tasmania, Hobart, Tasmania, Australia; 4 Hungarian Department of Biology and Ecology, Evolutionary Ecology Group, Babeş-Bolyai University, Cluj‐Napoca, Romania; 5 Department of Tisza Research, MTA Centre for Ecological Research-DRI, Debrecen, Hungary

## Abstract

The origin and subsequent maintenance of sex and recombination are among the most elusive and controversial problems in evolutionary biology. Here, we propose a novel hypothesis, suggesting that sexual reproduction not only evolved to reduce the negative effects of the accumulation of deleterious mutations and processes associated with pathogen and/or parasite resistance but also to prevent invasion by transmissible selfish neoplastic cheater cells, henceforth referred to as transmissible cancer cells. Sexual reproduction permits systematic change of the multicellular organism’s genotype and hence an enhanced detection of transmissible cancer cells by immune system. Given the omnipresence of oncogenic processes in multicellular organisms, together with the fact that transmissible cancer cells can have dramatic effects on their host fitness, our scenario suggests that the benefits of sex and concomitant recombination will be large and permanent, explaining why sexual reproduction is, despite its costs, the dominant mode of reproduction among eukaryotes.

## Abstract 2

The high prevalence (> 99%) of sexual reproduction among eukaryotes remains a major enigma in evolutionary biology. This Essay proposes that, in addition to previously suggested advantages, sexual reproduction also serves to prevent invasion by transmissible cancer cells that are potentially omnipresent among multicellular organisms.

One of the greatest enigma in evolutionary biology is the high prevalence (>99%) of sexual reproduction among eukaryotes [1,2]. Because sexual reproduction requires males that do not produce offspring, an asexual population should consequently reproduce faster than a sexual one [3]. Asexual individuals also benefit from maintaining co-adapted gene complexes and avoid costs involved in mate acquisition [4]. Despite this, the high prevalence of sexual reproduction in the natural world indirectly suggests that the selective forces behind the evolution of sex must be strong and pervasive.

Among the most prominent hypotheses that have been put forward to explain the evolution and maintenance of sexual reproduction, the Fisher–Muller hypothesis proposes that sex may rapidly generate multiple novel advantageous alleles [5–7]. Sexual reproduction will also reduce the deleterious effects of Muller’s ratchet, i.e., the build-up and accumulation of deleterious mutations in asexual organisms [8]. Another and probably the most famous hypothesis concerning the benefits of sexual reproduction suggests that recombination create novel genotypes that are able to resist pathogen and/or parasite infections (i.e., the Red Queen hypothesis) thereby maintaining host fitness despite endlessly evolving virulent pathogens/parasites [9,10]. Several empirical studies support this hypothesis [11–13], e.g., in facultative sexual crustacean *Daphnia magna*, sexually produced offspring were twice as resistant to parasites infecting the parents than asexual ones [14].

All the current hypotheses proposed to explain the evolution of sexual reproduction converge toward the idea that sexual reproduction is beneficial because the genetic diversity it creates provides significant evolutionary advantages to counteract infectious agents, enhance individual intra- and interspecific competition abilities, and alleviate the effects of ongoing fluctuations in environmental conditions [15]. However, what remains unclear is that occasional sex, rather than obligate, could presumably provide the above evolutionary benefits: according to most models, organisms that engage in sexual reproduction only sporadically seem to have the best of both worlds (e.g., [16,17]). Therefore, despite 50 years of research, the selective forces maintaining obligate sex are still not fully understood. Here, we argue that sex has been, and is still, favoured by selection because in contrast to asexual reproduction, it permits to reduce the fitness costs imposed by an ancestral enemy still present: transmissible malignant cell lines.

Multicellular organisms are societies of cooperating clonal cells that have emerged independently on several occasions approximately 1 billion years ago [18,19]. The primary benefit of multicellularity included the division of labor and specialization by differentiated cells [20]. The evolution of multicellular organisms, metazoans, required that individual cells had to forgo their own reproductive interests, i.e., shifting the Darwinian unit of selection from individual cells to the benefit of the entire multicellular community, i.e. the organism. However, one of the first challenges faced by asexual metazoans was, as for any cooperative system (e.g., [21]), the risk of exploitation by internal cheater cells, i.e., cancer cells [22]. Because uncontrolled proliferation of cancer cells is an ubiquitous phenomenon of metazoans [23], it has been proposed to have appeared during the transition from unicellularity to multicellularity [19]. Consequently, the first asexual multicellular organisms did not only have to deal with their own cheater cells but also to evolve adaptations preventing the colonization by infectious malignant cells coming from other individuals. Because anticancer defenses were presumably basic in the first multicellular organisms, both self and infectious cell lines were the major natural enemies. A mile stone in the evolution of metazoans was therefore to counteract and, if possible, to prevent the negative effects of internal cancer cells as well as those caused by non-self invaders, such as viruses, bacteria, parasites, as well as somatic and germ cell parasitism (e.g., in ascidians [24–26]) as well as transmissible cancer cells. These interactions ultimately resulted in the evolution of different evolved defense mechanisms (e.g., different branches and aspects of the immune system) across the animal kingdom. However, in order to reduce the deleterious effects of transmissible cancer cells, the metazoan immune system had to acquire an ability to differentiate between the former and healthy somatic cells.

While the ultimate fate of the vast majority of present malignant cancer cells is to perish with the death of their host, transmissible cancer cells have during the last decades been shown to occur in both invertebrates and vertebrates, often resulting in a massive increase of host mortality (reviewed in [27]). This transmission can involve direct routes such as interindividual aggression (e.g., biting), sexual interactions, and passive transport of transmissible cancer cells. The ability of some of these transmissible cancer cells to avoid immune recognition appears to emanate from a combination of reduced host genetic diversity (that could be the result of bottlenecks and small effective population size [28]) and an ability of the transmissible cancer to down-regulate their antigenic epitopes [27]. Other albeit rare transmissions of cancer cells have been observed in humans from mother to fetus [29,30], i.e., in which the maternally derived neoplastic cells in the infant had deleted human leukocyte antigen (HLA) alleles suggesting a possible mechanism for immune evasion [30]. Moreover, neoplastic leukemia cells arising in one monozygotic twin, having a single or monochorionic placenta, have been shown to transmit to the co-twin via intraplacental anastomoses [31,32], highlighting the impact of genetic similarity in the successful transmission of cancerous cells. Other organisms, such as the basal metazoan hydra, when reproducing asexually have also shown occurrence of vertical interindividual transmission of tumors [33].

Because clonal reproduction leads to organisms that are identical, we propose that (1) malignant cells produced by the first multicellular organisms were likely to be well adapted to other (identical) organisms, including direct descendants; (2) it was difficult for the victim organisms to recognize (and hence eliminate) transmissible cancer cells that were almost identical to normal somatic cells (i.e., immune evasion). An efficient way to prevent this was to be different from other individuals, and also to produce unique offspring. Organisms adopting sexual reproduction, conversely to clonal ones, form gametes, mix those together, and create progeny with an entirely novel genome. This both limits the chance for clonal infectious malignant cell lines to be already adapted to a novel host and increase the chance that victim organisms can immediately detect the colonization by a transmissible malignant cell, i.e., malignant cells are this time perceived as foreign allograft. Therefore, sexual reproduction could have evolved as an adaptive trait to prevent horizontal and/or vertical transmission of cancer cells (Fig 1).

**Fig 1:**
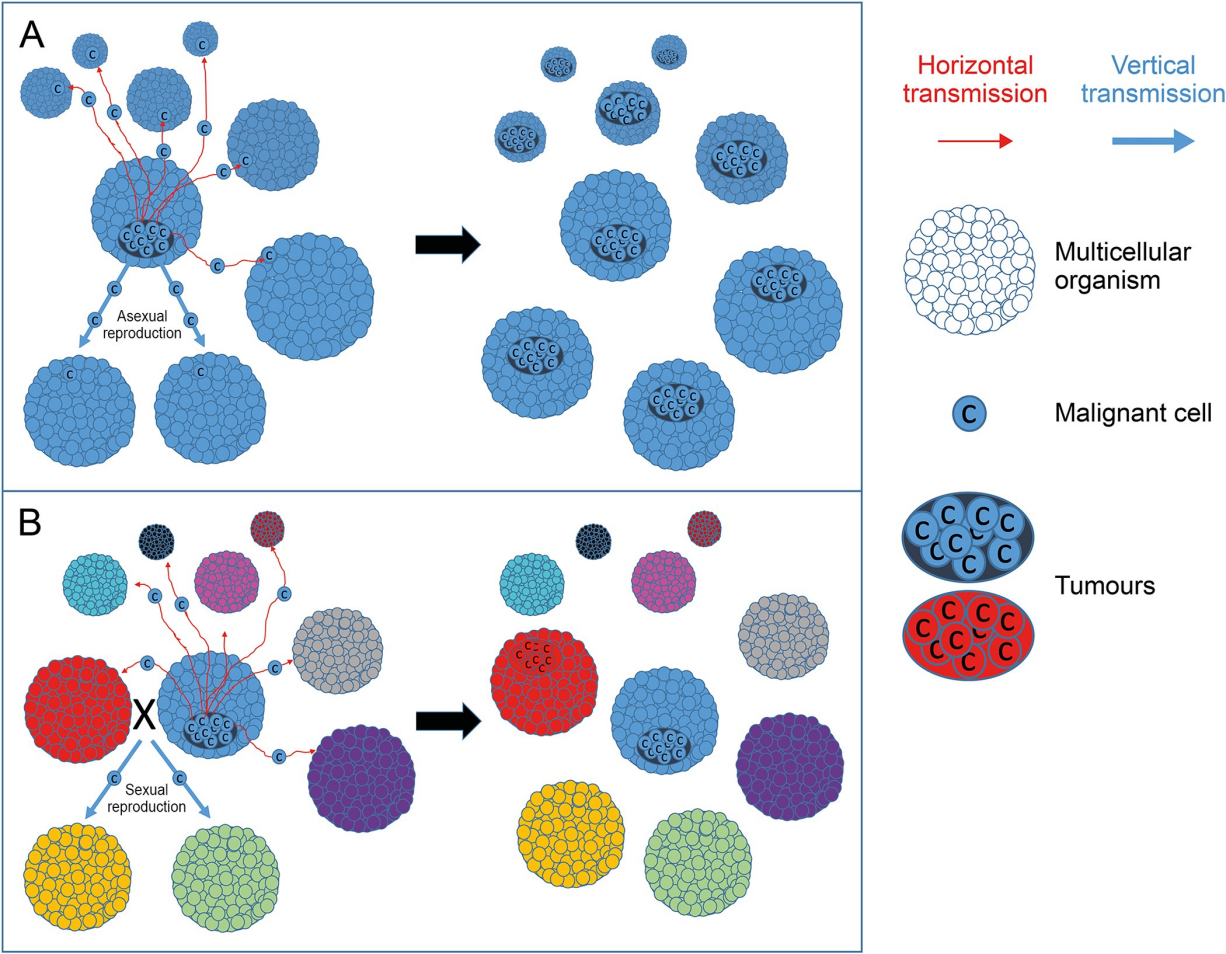
Asexual versus sexual reproduction and the transmission of malignant cells. (A) Asexual reproduction maintains high levels of interindividual similarity within a population, and this phenomenon increases the risk of vertical and horizontal transmission of malignant cells. (B) By blending genetics, sexual reproduction produces greater genetic diversity in a population, and as such, limits the transmission of cancer cells across individuals in the population. Genetic diversity facilitates the detection of the invading non-self cells and also limits the chances that the transmissible cancer cells are preadapted to the new host. Thus, cancer cells regularly emerge (e.g., red tumor) in individuals, but unless a “perfect storm” is present, as in the Tasmania devil/devil facial tumor disease system [27], malignant cells fail to be transmitted.

If the transmission of cancerous cells was a major factor in the evolution of sexual reproduction, the negative effects of such cheaters may also affect “super organisms,” such as social insect colonies, the queen being the gonads, the workers being the somatic cells, a system comparable to that of a multicellular organism [34]. Some social asexual ants and honey bees do develop asexual cheater workers that abandon reproductive self-restraint and reproduce at the expense of other colonies, hence adopting a behavior comparable to selfish transmissible cheater cells in a multicellular organism [35,36]. Moreover, analogous to transmissible cancerous cells, these cheater workers often invade other colonies with devastating consequences for the colony [35,36]. Just like host defense mechanisms (i.e., immune responses), the colonies of superorganisms police against the cheater workers, whereby the queen and/or the workers inhibit the reproduction of cheaters, by either attacking and mutilating recalcitrant workers or by consuming their eggs [37,38].

Empirical testing of theories that explain the evolutionary origin(s) of sex is often complex. However, several observations seem to support our hypothesis:

- Although sexual processes undoubtedly antedated multicellularity, no successful transition to multicellularity (i.e., when cancer emerged [33]) has avoided a tight connection with the sexual process [39,40]. Multicellularity may even set the stage for the overall diversity of sexual complexity throughout the Tree of Life [41].
- Species that are not affected by cancer, like prokaryotes and unicellular eukaryotes, should intermittently revert to asexual reproduction. Accordingly, bacteria and archaea reproduce primarily through asexual reproduction, usually by binary fission, with some genetic exchange and recombination occurring occasionally through horizontal gene transfer [42]. The majority of protists and fungi reproduce asexually via fissioning, budding, or spore production [43].
- Unlike animals, plants rarely develop cancer, potentially due to fundamental differences between plant and animal cellular structures, development, and physiology (reviewed in [44]). Plant cells possess rigid cell walls (containing hemicellulose fibers, pectin polysaccharides, and lignin) that maintain strict cellular structure and prevent uncontrolled cell growth. Plant cells also rarely accumulate enough mutations that would lead to cancer, due to their stem cells being hypersensitive to DNA damage and being ready for apoptosis in response to genetic abnormalities. The locomotion of tumor cells is also limited because plants rely on an acellular vascular system (i.e., the xylem and phloem), not on cellular circulatory systems such as blood or lymph vessels. Although plants can occasionally develop tumors, they occur much less often than in animals; they are not metastatic and certainly not as lethal [44,45]. Although plant reproductive strategies are highly diverse [46], many plants exhibit dual reproductive modes, producing both sexual and asexual offspring, being capable of vegetative reproduction (via rhizomes, runners, tubers, bulbils, etc.) and/or of asexual seed production [47,48].
- According to our theory, most asexual species should have a recent evolutionary history, whereas ancient asexual species should possess special adaptations to reduce the deleterious effects of cancer. Close to 50% of asexual lineages have been estimated to be <500,000 years old [49], whereas the remaining 50% of lineages consist of the “evolutionarily scandalous” organisms, such as orbatid mites, darwinulid ostracods, and bdelliod rotifers, that have persisted for millions of generations [49]. The latter species have indeed been shown to be resistant to mutagens such as radiation and heavy metals [50–53], which indicates high resistance to oncogenic processes and selection of tumor-suppressor mechanisms that enable the survival of these ancient asexual lineages.
  As a corollary, one might predict that recently evolved asexual species should be affected by cancer at higher frequency than their sexual conspecifics, unless they also have evolved efficient anticancer defenses. Further studies would be necessary to test these hypotheses.
- Multicellular eukaryotes that are strongly impacted by malignant cell emergence and proliferation should mostly have obligate sexual reproduction. Obligate sex is indeed the dominant mode of reproduction in many lineages of complex eukaryotes [1].
- Transmissible cancers should be rare in species practising sexual reproduction. Although we probably underestimate their prevalence [54], only 4 cases of transmissible cancers are currently known in the wild, supporting the idea that the evolution of transmissible cancer in sexually reproducing species is very rare and occurs only under very particular conditions (e.g., the “perfect storm hypothesis” [27]).

There are different possibilities to experimentally test our hypothesis. For instance, we predict that in organisms reproducing both by sexually and asexually, a shift toward more sexual reproduction should be observed following the emergence and progression of malignant cells. Hydra has the ability to switch between sexual and asexual reproduction and the propensity to develop tumors, therefore it could be a good candidate to test this hypothesis [33]. In accordance with our hypothesis, parental tumors are almost systematically transmitted to daughter polyps of hydra when reproduction is asexual (i.e., budding results in the vertical transmission of tumors), whereas offspring resulting from sexual reproduction are tumor free [33]. Demonstrating that tumor-bearing hydra, compared with healthy ones, preferentially reproduce sexually would provide support to our hypothesis. Because tumors can also be experimentally transplanted between polyps, this biological system also offers the possibility to test whether transplanted tumors establish better when recipient polyps are identical to the donor with tumors, compared with when they are different individuals.

Constant progress in animal cloning [55] could also help to evaluate the risk of cancer cell transmission associated with asexual reproduction. We predict that the likelihood of mother to fetus malignant cell transmission will be higher when the implanted embryos (e.g., in mammals) are genetically identical to their mother, compared with embryos that have originated form another female or are the mother’s natural embryos.

Comparative oncology approaches could also provide in depth analyses of the difference in anticancer defenses between recent and ancient asexual species, as well as in comparison with their sexual relatives. From a theoretical perspective, our hypothesis could be tested through developing new theoretical frameworks based on mathematical models so far used to elucidate the “Red Queen” hypothesis [56,57]. However, current mathematical models applied to host–pathogen interactions rarely consider parasite diversity. In the case of our hypothesis, future theoretical extensions will need to consider the fact that host diversity generated by sexual reproduction decreases the probability of cancer cell transmission and thus de facto reduces the diversity of the cancer cells that can be transmitted, which concomitantly allows the host’s immune system to be more efficient in eliminating them. Although including "parasite diversity” is generally mathematically challenging, combining recent theoretical developments in studying multistrain pathogens [56] with the Red Queen models would be an interesting first attempt.

## Concluding remarks

Although selfish neoplastic cells are omnipresent cheaters in all multicellular organisms [58], the among-individual transmission of such cells requires a “perfect storm” in sexual reproducing organisms with an optimal confluence of multiple host and tumor cell traits [27]. A major constraint of such transmissions requires an ability of transmissible cancerous cells to evade immunological histocompatibility barriers. Because asexual reproduction results in clonal, often identical organisms, asexual organisms and their progeny would be susceptible to the invasion of clonal transmissible cancer cells. Conversely, due to its enhanced among-individual genetic heterogeneity and concomitant increased ability to detect non-self cells, sexual reproduction should significantly reduce the risk of among-individual transmission of such cancerous cells. Given the ubiquity of oncogenic processes in the multicellular world together with the diversity of potential transmission routes, sexual reproduction, despite its associated costs, may consequently have been favored as a less risky, more profitable option to produce viable offspring, i.e., less subjective to transmissible cancers. To our knowledge, this selective scenario for the initial evolution of sex across the Tree of Life is novel. As illustrated, e.g., with the human twin example above, it also explains its continued maintenance despite the significant evolutionary costs. Also, the experimental approaches we proposed above should permit the evaluation of the critical role transmissible cancers play in shaping animal reproductive strategies.

In conclusion, we propose that the prevalence of sex in eukaryotes is a ghost of a past apogee of transmissible cancerous cell lines in the first asexual multicellular organisms. Although natural selection found a way to radically reduce the prevalence of transmissible cancer, sexual organisms are, however, still subjected to the deleterious effects of internal malignant cells. We hope that this paper will pave the way for a novel research direction on the evolutionary enigma of sex.

## Abbreviations

HLA: human leukocyte antigen
